# Common variable immunodeficiency (CVI): case report

**DOI:** 10.1186/1939-4551-8-S1-A268

**Published:** 2015-04-08

**Authors:** Luiz Carlos Bandoli Gomes

**Affiliations:** 1 Ufjf, Brazil

## Introduction

The CVI is the second most common primary immunodeficiency. The prevalence of CIV in the world is approximately 1:25,000. The age of presentation of ICV has peaks in the first decade if life and the beginning of the third decade. Both sexes are affected. Diagnostic criteria according to ESID/PAGID are: - recurrent infections; -age more 4 years; -reduced levels of IgG; decrease of IgA and/or IgM; exclusion of the other causes of hypogammaglobulinemia; - no isohemaglutininas and response to the vaccine. Five clinical phenotypes are described: only infections, autoimmunity, polyclonal lymphocytic infiltration, malignancy and enteropathy. The treatament of patients with CVI is performed with the use of an infusion of immunoglobulin.

## Objective

To report a patient with CVI associated with infections/ infestations recurrent and splenomegaly. Case description: H.S.M.A, male, 8 years old, eight pneumonia, low weight gain, infestation by Giardia lamblia and splenomegaly (physical examination and ultrasound).

## Tests

HIV ELISA negative. IgG=614mg/dl; IgM=19mg/dl; IgA=24mg/dl; CD4=24 (742mm3); CD8=70% (2150/mm3) and CD19=247/mm3. Lymphocyte B total=9.2%.

## Discussion

The CVI can be associated with some phenotypes, such as the occurrence of infections/infestations recurrent and splenomegaly. The levels of immunoglobulins are below the 3 percentile for age, the percentage of b lymphocytes in the minimum threstold, CD8 high.

## Conclusion

Reiterate the importance of the diagnosis of primary immunodeficiency in patients with infections/infestations recurrent splenomegaly and in this case the CVI by important prevalence.

## Consent

Written informed consent was obtained from the patient for publication of this abstract and any accompanying images. A copy of the written consent is available for review by the Editor of this journal.
